# Transpulmonary thermodilution in patients treated with veno-venous extracorporeal membrane oxygenation

**DOI:** 10.1186/s13613-021-00890-w

**Published:** 2021-07-02

**Authors:** Gregor Loosen, Alice Marguerite Conrad, Michael Hagman, Nils Essert, Manfred Thiel, Thomas Luecke, Joerg Krebs

**Affiliations:** 1 Department of Cardiothoracic Anaesthesia and Intensive Care, Royal Papworth Hospital NHS Foundation Trust, Papworth Road, Cambridge Biomedical Campus, Cambridge, CB2 0AY UK; 2 Department of Anaesthesiology and Critical Care Medicine, University Medical Centre Mannheim, Medical Faculty Mannheim of the University of Heidelberg, Theodor-Kutzer Ufer 1-3, 68167 Mannheim, Germany; 3 Department of Computational Linguistics, University of Heidelberg, Im Neuenheimer Feld 325, 69120 Heidelberg, Germany; 4 Interdisciplinary Centre for Scientific Computing, University of Heidelberg, Im Neuenheimer Feld 205, 69120 Heidelberg, Germany

**Keywords:** Acute respiratory distress syndrome, Extracorporeal membrane oxygenation, Transpulmonary thermodilution, Transesophageal echocardiography, Stroke volume measurement, Global end-diastolic volume, Extravascular lung water

## Abstract

**Background:**

We tested the effect of different blood flow levels in the extracorporeal circuit on the measurements of cardiac stroke volume (SV), global end-diastolic volume index (GEDVI) and extravascular lung water index derived from transpulmonary thermodilution (TPTD) in 20 patients with severe acute respiratory distress syndrome (ARDS) treated with veno-venous extracorporeal membrane oxygenation (ECMO).

**Methods:**

Comparative SV measurements with transesophageal echocardiography and TPTD were performed at least 5 times during the treatment of the patients. The data were interpreted with a Bland–Altman analysis corrected for repeated measurements. The interchangeability between both measurement modalities was calculated and the effects of extracorporeal blood flow on SV measurements with TPTD was analysed with a linear mixed effect model. GEDVI and EVLWI measurements were performed immediately before the termination of the ECMO therapy at a blood flow of 6 l/min, 4 l/min and 2 l/min and after the disconnection of the circuit in 7 patients.

**Results:**

170 pairs of comparative SV measurements were analysed. Average difference between the two modalities (bias) was 0.28 ml with an upper level of agreement of 40 ml and a lower level of agreement of -39 ml within a 95% confidence interval and an overall interchangeability rate between TPTD and Echo of 64%. ECMO blood flow did not influence the mean bias between Echo and TPTD (0.03 ml per l/min of ECMO blood flow; p = 0.992; CI − 6.74 to 6.81). GEDVI measurement was not significantly influenced by the blood flow in the ECMO circuit, whereas EVLWI differed at a blood flow of 6 l/min compared to no ECMO flow (25.9 ± 10.1 vs. 11.0 ± 4.2 ml/kg, p = 0.0035).

**Conclusions:**

Irrespectively of an established ECMO therapy, comparative SV measurements with Echo and TPTD are not interchangeable.

Such caveats also apply to the interpretation of EVLWI, especially with a high blood flow in the extracorporeal circulation. In such situations, the clinician should rely on other methods of evaluation of the amount of lung oedema with the haemodynamic situation, vasopressor support and cumulative fluid balance in mind.

*Trial registration*: German Clinical Trials Register (DRKS00021050). Registered 03/30/2020

https://www.drks.de/drks_web/navigate.do?navigationId=trial.HTML&TRIAL_ID=DRKS00017237

**Supplementary Information:**

The online version contains supplementary material available at 10.1186/s13613-021-00890-w.

## Background

Severe acute respiratory distress syndrome (ARDS) is a life-threatening organ dysfunction characterized by impaired lung mechanics, hypoxemia and hypercapnia [1]. Traditionally, patients are managed with mechanical ventilation, which may induce ventilator-associated lung injury [2, 3] resulting in increased morbidity and mortality [4, 5].

Recently, a systematic review showed a significant reduction in mortality in patients with most severe refractory respiratory failure treated with veno-venous extracorporeal membrane oxygenation (ECMO) compared to conventional treatment [6].

Arterial blood oxygen saturation and therefore oxygen delivery in these patients is the product of an interplay between circuit blood flow and blood flow through the native lungs, as the total venous return is divided between drainage into the femoral cannula and right atrium [7]. Hereby the blood leaving the oxygenator is fully oxygenated and has a high oxygen content whereas the fraction bypassing the extracorporeal circuit through the right heart and lungs is severely oxygen depleted as native lung does not contribute significantly to gas exchange in a highly acute situation of severe ARDS [7]. Hence, oxygen delivery depends on the cardiac output, the heart rate and stroke volume (SV) of the patient [8], the blood flow through the extracorporeal circuit and eventually on the ratio between the two blood flows [9, 10]. Therefore, knowledge of SV is desirable especially in patients who remain hypoxic because of intracardiac shunt [11] or hyperdynamic sepsis-induced vasoplegia and concomitant elevated CO [9, 12], despite sufficient ECMO support [13, 14]. Furthermore, an extracorporeal flow of greater than 60% of patient cardiac output ensures an arterial saturation above 90%, and therefore represents an important safety threshold [9]. Echocardiography (Echo) represents the gold standard to determine SV in patients treated with ECMO [15, 16], but needs formal education [17] and does not allow continuous SV measurement [18].

On the other hand, veno-arterial transpulmonary thermodilution (TPTD) is a reliable method for the measurement of SV and utilizes vascular catheters required anyways in the clinical management of ARDS [19]. Measurements are easily performed in patients treated without ECMO [18] and show a low interoperator variability [20]. Additionally, TPTD can quantify advanced volumetric parameters describing cardiac preload (global end-diastolic volume index, GEDVI) and lung oedema (extravascular lung water index, EVLWI) [19, 21] and facilitates continuous evaluation of SV combined with pulse contour analysis [22].

However, the effects of the extracorporeal circuit blood flow on TPTD measurement of SV, GEDVI and EVLWI are not well characterized. We hypothesize that blood flow in the ECMO circuit may be a major confounder of SV, GEDVI and EVLWI measurements utilizing TPTD.

Therefore, the aims of this study are:

1. To evaluate the interchangeability of SV measurement with Echo compared to TPTD in ECMO patients.
2. To evaluate the effects of blood flow in the extracorporeal circuit on SV measurements with TPTD.
3. To evaluate the effects of blood flow in the extracorporeal circuit on GEDVI and EVLWI measurements with TPTD.

## Methods

The study was approved by the local ethics committee (Medizinische Ethikkommission II, University Medical Centre Mannheim, Medical Faculty Mannheim of the University of Heidelberg, Mannheim, registration number 2019-719N-MA) and registered at the German Clinical Trials Register (DRKS00021050).

After obtaining written informed consent, we collected prospective data from 20 patients with severe ARDS managed with ECMO admitted to the Department of Anaesthesiology and Critical Care Medicine, University Medical Centre Mannheim, Medical Faculty Mannheim of the University of Heidelberg in Mannheim, Germany.

ECMO therapy was initiated according to standard operating procedures of the Department when patients fulfilled criteria published in the Extracorporeal Membrane Oxygenation for Severe Acute Respiratory Distress Syndrome trial [23].

Exclusion criteria for the study were: age younger than 18 years, contraindication against transesophageal probe insertion, pregnancy, inherited cardiac malformations or severe valvular dysfunction, end-stage chronic organ failure, expected survival of less than 24 h and therapy refractory haemodynamic instability (mean arterial pressure of less than 65 mmHg, heart rate of more than 130 beats/min or cardiac index of less than 2.0 l/min/m^2^).

SAPS II [24] and SOFA scores [25] were calculated for each patient at admission on the ICU. The RESP Score [26] was calculated just before ECMO cannulation. In all patients a 25 or 29 French drainage cannula (HLS Cannula, Maquet, Rastatt, Germany) and a 21 or 23 French return cannula (HLS Cannula, Maquet, Rastatt, Germany) were implanted in the femoral vein and in the internal jugular vein, respectively, to establish vascular access for the extracorporeal circuit. A magnetically levitated rotor pump (Centrimag Circulatory Support System, Abbot, GmbH, Wiesbaden, Germany) and gas exchange membrane (PLS System, Maquet, Rastatt, Germany) were used to complete the ECMO circuit.

All patients had a central venous catheter (inserted via the subclavian, internal jugular or axillary vein) in place, which was used for continuous drug infusion and TPTD. Additionally, we inserted a thermodilution catheter (4F/5F Pulsiocath™, Pulsion Medical Systems, Munich, Germany) in a femoral or brachial/axillary artery. A Pulse Contour Cardiac Output monitor (PiCCOplus™, Pulsion Medical Systems, Munich, Germany) was used for measurements of cardiac stroke volume, cardiac output (CO), GEDVI and EVLWI [27]. The thermodilution curve is segmented in the mean transit time (MTt) denoting the time point, when half of the applied thermoindicator has passed the arterial catheter and downslope time (DsT) denoting the exponential outwash of thermoindicator.

The device calculates GEDVI as

$$\frac{{{\text{CO}} \times {\text{MTt}} - {\text{CO}} \times {\text{DsT}}}}{{{\text{body}}\;{\text{surface}}\;{\text{of}}\;{\text{the}}\;{\text{patient}}\;({\text{m}}^{2} )}}$$

EVLWI is calculated as

$$\frac{{ - 0.25 \times {\text{CO}} \times {\text{MTt}} + 1.25 \times {\text{CO}} \times {\text{DsT}}}}{{{\text{body}}\;{\text{surface}}\;{\text{of}}\;{\text{the}}\;{\text{patient}}\; ({\text{m}}^{2} )}}$$

Patients were sedated with midazolam (5 to 15 mg/h) and sufentanil (0.5 to 2.5 µg/h) to achieve a Richmond Agitation–Sedation Score of -5 [28] and paralyzed with cisatracurium (6–20 mg/h) throughout each measurement [29]. Norepinephrine was used if mean arterial pressure was below 65 mmHg despite sufficient intravascular volume. Dobutamine was used if cardiac index was below 2.0 l/min/m^2^ despite sufficient cardiac pre- and afterload. Extracorporeal blood flow was titrated to achieve a partial pressure of arterial oxygen of at least 60 mmHg with ventilator (Engström Carestation, GE Healthcare, Munich, Germany) set to standardized respiratory settings (respiratory rate of 12/min, tidal volume of 2 ml/kg of ideal body weight, positive end-expiratory pressure according to the attending physician and an inspiration-to-expiration ratio of 1:1). These ventilator settings were used in every comparative SV measurement with Echo and TPTD.

During each measurement physiological values (haemodynamics, vasopressor dosage, intraabdominal pressure, gas exchange and the cumulative fluid balance) were noted.

### Experimental protocol

Comparative SV measurements with Echo and TPTD were performed at least 5 times during the treatment of each patient at 0–24 h, 24–72 h (1–3 days), 72–144 h (3–5 days), 144–216 h (5–7 days) and 216–288 h (7–9 days). Additional measurements were performed as clinically indicated by the attending physician.

### SV volume measurement with echocardiography

Transesophageal echocardiography (Vivid S4, GE healthcare, Solingen, Germany) was performed in left side positioning with 45 degree elevated upper body, according to recommendations of the American society of echocardiography [30]. In a mid-oesophageal long axis view (ME LAX 120°) the diameter of the left ventricular outflow tract (dLVOT) was measured and the cross-section area (CSA) was calculated as ${\text{CSA}} = \left( {\frac{{{\text{dLVOT}}}}{2}} \right)^{2} \times \pi$.

SV was calculated as CSA x mean VTI LVOT obtained from PW-Doppler in transgastric long axis view (TG LAX 110–130°) or deep transgastric five-chamber view (deep TG five-chamber 0°-20°). All TPTD measurements have been performed in the same positioning as TOE (45° upper body elevation).

VTI LVOT was measured over three full respiratory cycles. The intensivist performing the Echo was blinded for the results of TPTD. Echo measurements were performed by one of the authors (GL) and reviewed by a senior intensivist with expert knowledge in critical care echocardiography.

### Stroke volume measurement with transpulmonary thermodilution

SV measurement was obtained by TPTD simultaneously as the echocardiographic evaluation to ensure comparability. According to the recommendations of the manufacturer (PiCCOplus™, Pulsion Medical Systems SE, Munich, Germany) measurements were performed 3 times with 20 ml of cold saline (4 °C) and averaged. All TPTD measurements have been performed in the same positioning as the echocardiographic evaluation (45 degree upper body elevation).

### Measurement of GEDVI and EVLWI

We investigated the effects of different ECMO blood flows in patients with restored lung function who were no longer dependent on extracorporeal lung support on GEDVI and EVLWI measured with TPTD. Immediately before the termination of the ECMO therapy the blood flow was increased to 6 L per minute and GEDVI and EVLWI was measured with TPTD according to the protocol described above. Additional measurements were performed at a blood flow of 4 and 2 l/min and immediately after the extracorporeal circuit was removed from the patient.

### Statistical analysis

Statistical analyses were performed using SigmaPlot 11.0 (Systat Software GmbH, Erkrath, Germany) and R 3.3.1 (R Core Team, 2016) by a dedicated statistician (MH). The level of significance was set at *p* < 0.05. We hypothesized that blood flow in the ECMO circuit would be a major confounder of SV measurements utilizing TPTD. So, for analysis purposes SV measurements were binned according to ECMO flow (2.0–2.9 l/min, 3.0–3.9 l/min, 4.0–4.9 l/min and > 5.0 l/min).

Precision of echocardiographic measurement was calculated as follows [31]: the coefficient of variation (CV) is calculated as the SD of measurements divided by the mean. By dividing by the number of measurements the coefficient of error (CE) is derived. Precision is then two times CE. The least significant change (LSC) between two measurements has been defined as.

${\text {LSC}} = {\text{CE}} \times 1,96 \times \sqrt{2}$ [31]. According to Jozwiak et al., we calculated LSC for intra-examination analysis between measurements of the first and the third respiratory cycle [32].

A Bland–Altman plot was used for graphical visualization of the agreement between Echo and TPTD [33]. Bias was calculated as the mean of the differences of both measurement modalities.

95% limits of agreement (LOA) were calculated as mean difference ± 1.96 times the difference of variances of repeated differences between the two methods on the same subject and the differences between the averages of the two methods across subjects corrected by the number of observations on each subject as proposed by Bland et al. [34] for repeated measurements with varying true values.

The percentage error was calculated as 1.96 times the standard deviation of the difference of both measurement techniques divided by the mean SV of both methods [35].

Furthermore, we performed an interchangeability analysis as proposed by Lorne et al. based upon a calculation of the repeatability coefficient for our Echo measurements.

Briefly, a pair of SV measurements with Echo and TPTD is deemed interchangeable when the absolute difference between both measurements is lower or equal to the maximum difference, which would result from repeating the measurement with the reference method.

The repeatability coefficient represents the internal validity of the Echo measurement itself was calculated for each measurement and then averaged (170 repeatability coefficients, each for every measurement). Mathematically, this is described by the repeatability (R) of the reference method [36]:

$$R_{{{\text{SV \; Echo}}}} = \sqrt {\frac{{\sum ({\text{SV \; Echo}}_{1} - {\text{SV \; Echo}}_{2} )^{2} }}{n}}$$

and the repeatability coefficient (RC), which is defined as:

$${\text{RC}}_{{{\text{SV \; Echo}}}} = 1,96 \times \frac{{R_{{{\text{SV \; Echo}}}} }}{{{\text{mean}}\;{\text{of}}\;{\text{data}}}}$$

Therefore, the maximum acceptable difference between Echo and TPTD, in order to count as interchangeable is:

Interchangeability = $$\frac{{{\text{SV}}_{{{\text{Echo}}}} {\text{ + SV}}_{{{\text{TPTD}}}} }}{2} \times \sqrt {({\text{RC}}_{{{\text{SV \; Echo}}}} )^{2} + ({\text{RC}}_{{{\text{SV \; Echo}}}} )^{2} }$$

Furthermore, we conducted a subgroup analysis regarding the central venous catheter insertion side including Bland–Altman plots and the interchangeability.

Data comparing SV in distinctive ECMO flow ranges are shown as individual measurements and median. We used a linear mixed effect model (LMEM) with a fixed effect for flow and a subject-specific random component for intercept and flow to analyse the influence of ECMO circuit flow on the bias between Echo and TPTD measurement.

Data describing the influence of the ECMO blood flow on EVLWI and GEDVI measurements are shown as mean ± standard deviation. In both instances analysis of variance followed by Holm–Sidak’s post hoc test was used.

## Results

In the study period from 03/2020 to 06/2020 65 patients with severe cardiopulmonary dysfunction were admitted to ICU in the study period. 37 of them were managed without ECMO, 3 were managed with veno-arterial or veno-arterio-venous ECMO and 2 patients died within less than 24 h. A patient flowchart is presented in Additional file 1: Fig. S1. In the remaining 20 patients, we performed 186 comparative cardiac stroke volume measurements with Echo and TPTD. 16 measurement pairs were excluded from the analysis because patients presented arrhythmia of various types, resulting in 170 analysed comparative measurements. A measurement flowchart is presented in Additional file 1: Fig. S2.

Table 1 shows anthropometric characteristics, the initial cause of ARDS and the length of ICU stay of the patients. Overall mortality was 35%. Mean SAPS II, SOFA and RESP scores are presented in Table 1. Physiological values (ventilator settings, haemodynamics, vasopressor dosage, intraabdominal pressure, gas exchange and the cumulative fluid balance) grouped according to ECMO blood flow are presented in Additional file 1: Table S2.

**Table 1:** Anthropometric characteristics for all patients

	All (n = 20)
Sex (male/female)	11/9
Age (years)	57 ± 11
Type of ARDS	
Community acquired pneumonia	
Covid 19 pneumonia	8
Pneumococcal pneumonia	3
Other	2
Ventilator associated pneumonia	2
Anastomotic leakage	3
Pancreatitis	2
ICU stay [d]	27 ± 10
SAPS II	61 ± 13
SOFA	11 ± 3
RESP	2 ± 4

ARDS, acute respiratory distress syndrome; ICU, intensive care unit; SAPS II, Simplified Acute Physiology Score II; SOFA, Sepsis-related Organ Failure Assessment score; RESP, Respiratory Extracorporeal Membrane Oxygenation Survival Prediction (RESP) score

**Fig. 1:**
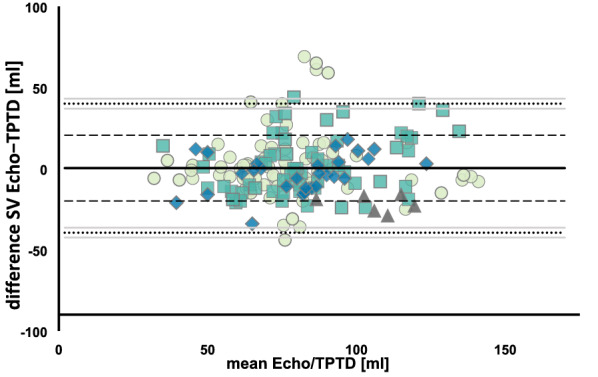
Bland–Altman plot showing bias and LOA between the SV measurements with TPTD and echocardiography. Data are expressed as mean between the SV measurements of TPTD and echocardiography plotted against the difference of both measurements. Bias = 0.28 (black line), upper level of agreement = 40 ml, lower level of agreement = − 39 ml within a 95% confidence interval (dotted line) mint circle, ECMO blood flow 2.0–2.9 l/min; green square, ECMO blood flow 3.0–3.9 l/min; blue rhombus, ECMO blood flow 4.0–4.9 l/min; grey triangle; ECMO blood flow > 5.0 l/min. Echo, echocardiography; TPTD, transpulmonary thermodilution; SV, stroke volume

Average precision of echocardiography was 2.4 (1.8–2.7) %, with a CV of 5.6 (3.6–6.1) % and the inter-observer LSC was calculated as 5.5 (3–5.6) %. Figure 1 shows the resulting Bland–Altman plot derived from paired Echo and TPTD measurements of SV. The average difference between the two modalities (bias) was 0.28, the upper level of agreement was 40 ml and the lower level of agreement was -39 ml within a 95% confidence interval, respectively. The percentage error was 45%.

**Fig. 2:**
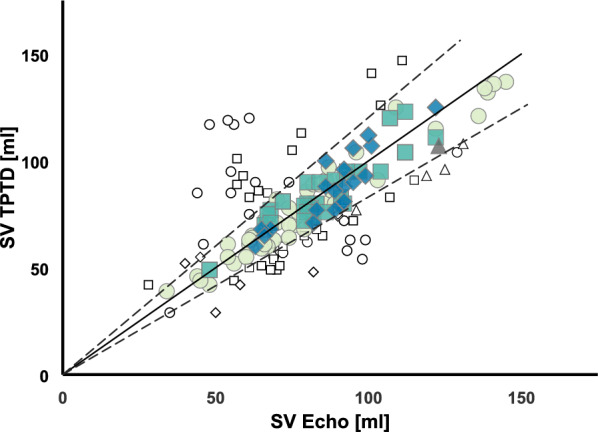
Interchangeability analysis. Interchangeability analysis with corresponding interchangeability sector (dashed lines) mint circle, ECMO blood flow 2.0–2.9 l/min; green square, ECMO blood flow 3.0–3.9 l/min; blue rhombus, ECMO blood flow 4.0–4.9 l/min; grey triangle; ECMO blood flow > 5.0 l/min white symbols denote not-interchangeable comparative measurements between Echo and TPTD, colours denote interchangeable pairs of measurements. Echo, echocardiography; TPTD, transpulmonary thermodilution; SV, stroke volume

**Fig. 3:**
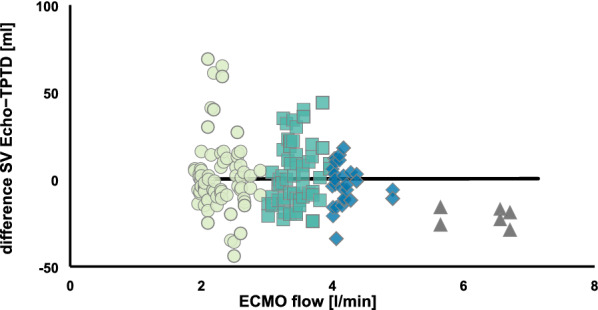
Influence of ECMO blood flow on SV measurements with TPTD. Influence of ECMO blood flow on SV measurements with TPTD analysed with a linear mixed model mint circle, ECMO blood flow 2.0–2.9 l/min; green square, ECMO blood flow 3.0–3.9 l/min; blue rhombus, ECMO blood flow 4.0–4.9 l/min; grey triangle; ECMO blood flow > 5.0 l/min median, black line. Echo, echocardiography; TPTD, transpulmonary thermodilution; SV, stroke volume; ECMO, extracorporeal membrane oxygenation

The overall interchangeability rate of TPTD and Echo was 64% based upon a repeatability coefficient for Echo measurements of 0.13 (Fig. 2). The results were graphically visualized by a corresponding interchangeability sector. Mean ECMO blood flow during all measurements was 3.2 ± 0.9 l/min. As shown in Fig. 3, ECMO blood flow had no significant influence on the mean bias between Echo and TPTD (0.03 ml per l/min of ECMO blood flow; p = 0.992, CI -6.74 – 6.81).

We performed a post hoc analysis evaluating the effects of the venous catheter insertion site (internal jugular vein, axillary vein, subclavian vein) on the Bland–Altman and interchangeability analysis. 96 comparative SV measurements with TPTD and Echo with a central venous catheter in the internal jugular vein, 48 in the axillary and 26 in the subclavian vein are included in the analysis (Additional file 1: Figs. S3–S8, Table S1). We found no statistical relevant differences on the bias between the three different catheter placements. The percentage errors were 38.9% (internal jugular vein), 34.3% (axillary vein) and 76.4 (subclavian vein). The interchangeability rates were 60% (internal jugular vein), 69% (axillary vein) and 23% (subclavian vein).

Additionally, we compared measurements of advanced volumetric parameters by TPTD with an ECMO flow of 6, 4, 2 and 0 l/min immediately after ECMO therapy termination in 7 patients. Non-survivors were not eligible for the sub-study (7 patients). In 6 patients we could not perform the measurements because of logistic reasons and non-availability of study team.

We found no significant differences of GEDVI irrespectively of the blood flow in the ECMO circuit. EVLWI on the other hand, differed significantly between measurements at a blood flow of 6 l/min and those without ECMO flow (25.9 ± 10.1 vs. 11.0 ± 4.2 ml/kg, p = 0.0035) (Table 2). Cardiac output was consistent over all ECMO flow ranges and without extracorporeal flow (p = 0.364) (Table 2).

**Table 2:** Haemodynamics, GEDVI and EVLWI of seven patients at different ECMO blood flows

	ECMO blood flow 0 l/min	ECMO blood flow 2 l/min	ECMO blood flow 4 l/min	ECMO blood flow 6 l/min
Stroke volume (ml)	94.4 ± 24.6	106.3 ± 50.5	85.9 ± 26	85.3 ± 31.7
Heart rate (1/min)	72.4 ± 14.4	75.9 ± 19.6	76.4 ± 21.3	73.2 ± 17.0
Cardiac output (l/min)	6.7 ± 1.5	7.6 ± 2.7	6.5 ± 2.1	6.0 ± 1.7
GEDVI (ml/m^2^)	641.7 ± 126.7	686.3 ± 191.3	551.9 ± 144.7	549.3 ± 165.8
EVLWI (ml/m^2^)	11.0 ± 4.2	13.0 ± 8.3	17.5 ± 10.8	25.9 ± 10.1^a^

GEDVI, global end-diastolic volume index; EVLWI, extravascular lung water index

^a^ECMO blood flow 0 l/min vs. ECMO blood flow 6 l/min, p = 0.0035

## Discussion

The main findings of this study can be summarized as follows:

1. SV measurements of Echo and TPTD are not interchangeable from a clinical point of view which is in line with previously reported results.
2. ECMO circuit flow had no influence on the bias between SV measurement by Echo and TPTD.
3. GEDVI measurements were consistent across the entire range of extracorporeal blood flow. In contrast, EVLWI was influenced by the ECMO circuit.

### SV measured by TPTD in patients treated with ECMO

There is an ongoing discussion about comparative SV measurements with TPTD and Echo [35, 37], mostly around the question which amount of discrepancy between both modalities is considered acceptable.

In their meta-analysis Critchley and Critchley proposed to accept a percentage error of 30% between two techniques measuring SV [38]. This proposal assumed an intra-individual precision error of 20% compared to the real cardiac output. Their work has been criticized as clinically irrelevant [39] for its reliance on results from the controlled environment of the cardiac catheterization laboratory, in vitro simulations [40, 41] and for the inclusion of data from animal studies [42, 43]. A systematic review from Wetterslev et al. reports percentage errors between 11 and 69% comparing various echocardiographic and transpulmonary measurement techniques [35].

We found a percentage error of 45% comparing Echo and TPTD, which is in line with the findings of Botero et al. [44] comparing carbon dioxide rebreathing, bolus and continuous thermodilution with the transit time flow probe method as proposed gold standard. They reported percentage errors between 37.3% and 64.7%. Graeser et al. compared cardiac output measured by 3D Echo with thermodilution and noted a wide limit of agreement and a percentage error of 55% [45]. In a meta-analysis by Peyton et al. analysing 47 studies the authors reported a similar range of accuracy and precision of oesophageal Doppler, partial carbon dioxide rebreathing, transthoracic bioimpedance and pulse contour analysis [46]. So, overall, our results are within the range reported by the literature mentioned above.

Inter-examination analysis of echocardiographic measurements can be performed by calculating the CV, precision and LSC [32]. Our results are in line with previously reported CV [47, 48]. As our study protocol averaged SV over three respiratory cycles and therefore a high number of total SV have been measured, the mathematical precision is low, again those results are comparable to transthoracic SV measurements conducted in a big cohort of intensive care patients by Jozwiak et al. [32]. This study group also investigated on the LSC and found a LSC of 11 (5–18) %, which is slightly higher than our result of 5.5 (3–5.6) %. This might be due to the differences in study protocol with two changes of examiners as performed by Jozwiak et al. and consequent time delay or slight dislocation of the probe, and acquisition of all measurements by a single physician during one examination in our study.

Limitations of a mere Bland–Altman plot and percentage error analysis have been demonstrated [36, 49], particularly the levels of agreement do not necessarily reflect the actual clinical usefulness of either method.

To address this issue, we conducted an interchangeability analysis as previously described by Lorne et al. [36]. We calculated a repeatability coefficient of 0.13 (13%, respectively) from the Echo measurements that is well in line with reported values between 8.6% [48], 12.6% [47] and 16% [50]. Other Echo parameters addressing left ventricular function report repeatability values ranging from 10.7 to 35.6% [51]. Whether two modalities of measurement of the same parameter are interchangeable or not is strongly dependent on precision of the reference method. Hypothetical implementation of the reported 16% leads to an interchangeability rate of 74% and by implementing 20% precision as earlier proposed by Critchley and Critchley 84% of paired measurements are deemed interchangeable.

Unfortunately, to our knowledge no study conducted such interchangeability analysis for patients without ECMO for Echo and TPTD measurements, and predominantly Bland–Altman analyses were reported throughout meta-analyses and reviews [35, 52]. In their original publication Lorne et al. calculated an interchangeability rate of approximately 65% for comparative cardiac output measurements acquired with pulmonary artery thermodilution and TPTD, which they deemed clinically irrelevant [36].

Therefore, we can only conclude that our results are within the margin outlined by percentage error comparisons conducted in patients without ECMO but cannot draw any direct conclusions as to the clinical usefulness. Interchangeability rate in patients without ECMO between Swan–Ganz Catheter and TPTD have been reported to be around 60% [36], which is also in line with our findings.

Traditionally, TPTD is not recommended in patients with veno-venous ECMO because of potential recirculation of indicator into the extracorporeal circuit [53, 54], which might lead to systematic overestimation of SV [55]. Recirculation depends on a variety of other factors like total ECMO flow, reinfusion pressure and especially cannula design, diameter and position [56, 57]. Broman et al. investigated pressure and flow properties of various cannula types and diameters in an ex vivo experiment. One of their main findings was a significant reduction of negative drainage pressure gradients with multi stage cannulas with larger diameter and thus a reduction of recirculation [58]. Therefore, as part of a therapeutic strategy to minimize recirculation our institution generally implants large bore drainage and reperfusion cannulas.

The quantification of recirculation is challenging in clinical practice because it requires measurement of the central venous oxygen saturation without the admixture of oxygenized blood from the ECMO circuit [59]. Alternatively, turning off the sweep gas flow on the ECMO membrane as proposed by van Heijst et al. [60] might be potentially harmful in a condition where maximal lung support is required [61] and thus we opted not to quantify recirculation in this study.

It should be noted that, the mathematical algorithm for the measurement of SV utilized by the PiCCOplus™ takes into account that even under physiological conditions without ECMO a certain amount of recirculation is present and deforms the declining part of the TPTD curve accordingly. Therefore, SV is calculated by a corrected thermodilution curve [62]. So, in theory the effect of indicator recirculation in the ECMO circuit on SV measurement might be mitigated by the algorithms used by the PiCCOplus™ thermodilution monitoring system.

Herner et al. compared SV measurement using TPTD in patients before and after ECMO insertion [63]. Unlike the results of a small study by Haller et al. [54], where cardiac output was overestimated to a maximum of 300%, they found no significant alterations of SV measurement before and after ECMO initiation. Furthermore, they found no influence of the injection side (jugular vs. femoral) of the thermoindicator. This is contrary to our findings in the post hoc subgroup analysis of the effects of different central venous catheter insertion sides. The percentage error was higher and the interchangeability rate lower when a subclavian vein catheter was used for SV measurements with TPTD. Interestingly, Yu et al. found no significant difference in SV but on advanced haemodynamic parameter measurements with TPTD between regular subclavian injection and subclavian catheter misplaced in the internal jugular vein in patients without ECMO [64].

On the other hand, this study is limited by the fact that the time interval between the two measurements was substantial (13.5 ± 9.3 h), so it remains unclear whether the patients were exactly in the same cardiocirculatory condition during both measurements.

As the mean bias between both measurement modalities in our study is rather small, we believe that there was no systematic overestimation of SV when comparing simultaneous measurements with both methods, implying either the absence of significant recirculation or relevant indicator loss in our ECMO setup. Furthermore, there is no visual trend of decreasing interchangeability attributable to increasing ECMO flow.

As demonstrated in our study, in our setting EMCO blood flow does not affect the mean bias between SV measured by Echo and TPTD. Therefore, the same considerations comparing Echo and TPTD as in a patient treated without an extracorporeal circuit apply. Referring to the interpretation of Lorne et al. [36], these SV measurements are not interchangeable.

### Measurement of GEDVI and EVLWI during and after ECMO

Because of the aforementioned potential recirculation of indicator in the circuit, there are concerns regarding the reliability of the measurement of GEDVI and EVLWI in patients treated with ECMO [54]. Recirculation of indicator tends to mainly influence DsT but not the MTt of the temperature curve assessed by the PiCCOplus™ thermodilution monitor [65].

A prolongation of DsT corresponds to an increased EVLWI as it indicates the exponential outwash of thermoindicator [66, 67].

This was first observed in patients with intracardiac left-to-right shunt where EVLWI increased and GEDVI decreased [68, 69] because of indicator circulation between both atria. Schmidt et al. described a prolonged DsT in TPTD and increased EVLWI measurement in patients treated with continuous renal replacement therapy, which was dependent on the blood flow in the extracorporeal circuit [67].

Herner et al. reported elevated EVLWI after ECMO initiation, although in a multiple regression analysis only central venous line placement in the femoral vein but not blood flow was associated with an increase in EVLWI. They concluded that EVLWI, and to a lesser extent GEDVI, might be more prone to measurement alterations due to recirculation of thermoindicator in the extracorporeal circuit. This was emphasized by a significant increase of DsT after initiating of the ECMO therapy in their study. As expected, with a blood flow of 6 l/min circuit, we found a significant increase of EVLWI compared to no ECMO flow. As we were not able to measure thermoindicator recirculation and the resulting prolonged DsT, we can only speculate about the cause of our observations, though.

Furthermore, we found no statistical differences in GEDVI measurement regardless of blood flow in the ECMO circuit. Again, these results are in line with Herner et al., who reported no alteration in GEDVI before and after ECMO initiation in patients with a central venous line in the jugular vein [63]. As MTt denotes the time point, when half of the applied thermoindicator passes the arterial catheter and unlike to DsT, which can be prolonged substantially by multiple passes of the thermoindicator through the extracorporeal circuit, MTt is only influenced by indicator loss during the first pass through the circuit [63]. Therefore, Herner et al. concluded, that although they found a significant increase in MTt, the absolute value of this increase was not large enough to significantly influence GEDVI measurements [63]. On the other hand, the impact of DsT on EVLW measurement is higher than MTt as described by the underlying mathematical equation. Therefore, a change in MTt is likely to have a more pronounced impact on GEDVI than on EVLWI measurement.

### Limitations

There is no clinically available gold standard method for measurement of SV especially in patients treated with ECMO [16]. As Echo is widely used in high volume centres, we aimed to compare TPTD with Echo in these patients, albeit there being an ongoing discussion regarding the feasibility, usability and interoperator reliability [70, 71] of Echo based SV measurement in critically ill patients. Therefore, the use of Echo as our reference method might be debatable. On the other hand, our data showed the expected precision as reported by the literature [35, 44–48, 50]. We excluded patients without a sinus rhythm from our analysis. Because of this, we can draw no conclusions of the comparability and interchangeability of SV and advanced haemodynamic measurements in arrhythmic patients.

Furthermore, we performed repeated measurements in a limited number of patients. Generally speaking, a sample size smaller than 20 is usually not recommended for a Bland–Altman analysis is applied [49]. It has also been stated that as long as repeated measurements per individual are fewer than individuals the changes in Bland–Altman analysis (bias, LOA) will not be clinical apparent [34]. Therefore, we choose a practical approach, taking those aspects into account: a patient number of 20 to fulfil the first mentioned recommendation with a minimum of 5 measurements per participant to get to a total number of 100 paired measurements which results in 95% CI about ± 0.34 SD.

Additional measurements were performed as clinically indicated and even with doubling the number of measurements per patient the second requirement of keeping numbers of measurements per patient significant below number of total participants would not have been violated.

In our post hoc analysis evaluating the effects of the venous catheter insertion site we found profound differences in the measurement of cardiac stroke volume with a subclavian and an internal jugular, respectively, axillary vein catheter in the Bland–Altman and interchangeability analyses. Percentage error and interchangeability between the placement of the catheter in the subclavian vein and the internal jugular, respectively, axillary vein differ obviously and are clinically relevant. The interpretation of our results of the subgroup analysis merits careful consideration as it was not the aim of the study to differentiate the effects of venous catheter insertion sites on SV and advanced haemodynamic measurements in ECMO patients.

Our results concerning GEDVI and EVLWI are in line with findings of other author [72], but because of the limited number of patients eligible for those measurements, we can only speculate about the underlying mechanisms. We therefore interpret our data as hypothesis generating. Besides the low patient numbers included in our analysis we would emphasize, that we investigated the effects of the blood flow of the circuit at six, four, two and immediately before the termination with a of the ECMO therapy with no blood flow. The whole sub-study took about 20 min, so it is unlikely that there was a profound change in GEDVI or EVLWI per se.

## Conclusion

Measuring SV with TPTD is easily performed even by untrained physicians. The data derived from these measurements in patients treated with ECMO are precise regardless of the blood flow in the ECMO circuit. Percentage error and interchangeability are within the range of data from patients without extracorporeal membrane oxygenation. Therefore, the attending clinician at the bedside has to accept the same limitations when comparing SV measurements with Echo and TPTD. Our data imply, that, irrespectively of an established ECMO therapy, comparative SV measurements with Echo and TPTD are not interchangeable accordingly to the criteria defined by Lorne et al. [36].

Such caveats also apply to the interpretation of EVLWI, especially with a high blood flow in the extracorporeal circulation. In such situations, the clinician should rely on other methods of evaluation of the amount of lung oedema with the haemodynamic situation, vasopressor support and cumulative fluid balance in mind.

## Supplementary Information

**Additional file 1**.

## Data Availability

The datasets used and/or analysed during the current study are available from the corresponding author on reasonable request.

## Abbreviations

ARDS: Acute respiratory distress syndrome
CO: Cardiac output
CSA: Cross-sectional area
(d)LVOT: (Diameter of the) left ventricular outflow tract
DsT: Downslope time
Echo: Echocardiography
ECMO: Extracorporeal membrane oxygenation
GEDVI: Global end-diastolic blood volume index
ME LAX: Mid-oesophageal long axis (view)
MTt: Mean transit time
R: Repeatability
RC: Repeatability coefficient
RESP: Respiratory Extracorporeal Membrane Oxygenation Survival Prediction Score
SAPS II: Simplified Acute Physiology Score II
SOFA: Sequential Organ Failure Assessment
SV: Stroke volume
TG LAX: Transgastric long axis (view)
TPTD: Transpulmonary thermodilution
VTI: Velocity time integral
