# Supplementary material for: Transpulmonary thermodilution in patients treated with veno-venous extracorporeal membrane oxygenation
Source: Ann Intensive Care. 2021 Jul 2;11:101. doi: 10.1186/s13613-021-00890-w (PMC8249841; doi:10.1186/s13613-021-00890-w)
Supplement: Supplementary file 1 — Additional file 1. [file 13613_2021_890_MOESM1_ESM.docx]

**Figure 1:** patient flow chart

65 patients admitted to ICU with severe cardiopulmonary dysfunction

37 patients managed conservatively

3 patients managed with VA/VAV-ECMO

2 patients died in less than 24 hours

3 patients without informed consent

20 patients with VV-ECMO included in the study

**Figure 1:** patient flow chart

ICU, *intensive care unit*; ECMO, *extracorporeal membrane oxygenation*; VA-ECMO, *veno-arterial ECMO*, VAV-ECMO, *veno-arterio-venous ECMO*

**Figure 2:** measurement flow chart

20 patients with VV-ECMO included in the study

16 SV measurements with arrhythmia

170 SV measurements comparing TPTD and echocardiography with sinus rhythm

7 patients died

6 patients measurement not feasible

Measurement of GEDVI and EVLWI at ECMO blood flow of six, four, two and zero blood flow in 7 patients

**Figure 2:** measurement flow chart

SV, *cardiac stroke volume*; TPTD, *transpulmonary thermodilution*

**Figure 3:** Bland-Altman-Plot of SV measurements by transpulmonary thermodilution and echocardiography with the central venous catheter inserted in the internal jugular vein

**Figure 3:** Bland-Altman-Plot of cardiac stroke volume measured by transpulmonary thermodilution and echocardiography with the central venous catheter inserted in the internal jugular vein

Data is expressed as mean between the SV measurements of TPTD and echocardiography plotted against the difference of both measurements

Bias = -2.3 ml (black line), upper level of agreement = 27.8 ml, lower level of agreement = - 32.5 ml (dashed lines), percentage error: 38.9 %

Echo, *echocardiography;* TPTD, *transpulmonary thermodilution;* SV*, stroke volume*

**Figure 4:** Interchangeability analysis with corresponding interchangeability sector (dashed lines) with the central venous catheter inserted in the internal jugular vein

**Figure 4:** Interchangeability analysis with corresponding interchangeability sector (dashed lines) with the central venous catheter inserted in the internal jugular vein

Interchangeability rate: 60%

Echo, *echocardiography;* TPTD, *transpulmonary thermodilution;* SV*, stroke volume*

**Figure 5:** Bland-Altman-Plot of SV measurements by transpulmonary thermodilution and echocardiography with the central venous catheter inserted in the axillary vein

**Figure 5:** Bland-Altman-Plot of SV measurements by transpulmonary thermodilution and echocardiography with the central venous catheter inserted in the axillary vein

Data is expressed as mean between the SV measurements of TPTD and echocardiography plotted against the difference of both measurements

Bias = 1.9 ml (black line), upper level of agreement = 32.3 ml, lower level of agreement = -28.5 ml (dashed lines), percentage error: 34.3 %

Echo, *echocardiography;* TPTD, *transpulmonary thermodilution;* SV*, stroke volume*

**Figure 6:** Interchangeability analysis with corresponding interchangeability sector (dashed lines) with the central venous catheter inserted in the axillary vein

**Figure 6:** Interchangeability analysis with corresponding interchangeability sector (dashed lines) with the central venous catheter inserted in the axillary vein

Interchangeability rate: 69%

Echo, *echocardiography;* TPTD, *transpulmonary thermodilution;* SV*, stroke volume*

**Figure 7:** Bland-Altman-Plot of SV measurements by transpulmonary thermodilution and echocardiography with the central venous catheter inserted in the subclavian vein

**Figure 7:** Bland-Altman-Plot of SV measurements by transpulmonary thermodilution and echocardiography with the central venous catheter inserted in the subclavian vein

Data is expressed as mean between the SV measurements of TPTD and echocardiography plotted against the difference of both measurements

Bias = 6.5 ml (black line), upper level of agreement = 68.2 ml, lower level of agreement = -55.1 ml (dashed lines), percentage error: 76.4 %

Echo, *echocardiography;* TPTD, *transpulmonary thermodilution;* SV*, stroke volume*

**Figure 8:** Interchangeability analysis with corresponding interchangeability sector (dashed lines) with the central venous catheter inserted in the subclavian vein

**Figure 8:** Interchangeability analysis with corresponding interchangeability sector (dashed lines) with the central venous catheter inserted in the subclavian vein

Interchangeability rate: 23%

Echo, *echocardiography;* TPTD, *transpulmonary thermodilution;* SV*, stroke volume*

**Table 1:** summary of the post hoc analysis of the effects of venous catheter placement on comparative cardiac stroke volume measurement with echocardiography and transpulmonary thermodilution

|  | **internal jugular vein** | **axillary vein** | **subclavian vein** |
| --- | --- | --- | --- |
|  | n = 96 | n = 48 | n = 26 |
|  |  |  |  |
| **percentage error [%]** | 38.9 | 34.3 | 76.4 |
| **interchangeability rate [%]** | 60 | 69 | 23 |
| **difference in mean [ml]** | -2.3 | 1.9 | 6.5 |

**Table 1:** summary of the post hoc analysis of the effects of venous catheter placement on comparative cardiac stroke volume measurement with echocardiography and transpulmonary thermodilution

**Table 2:** physiological data

|  | **ECMO flow 2.0 - 2.9 [liters/min]** | **ECMO flow 3.0 - 3.9 [liters/min]** | **ECMO flow 4.0 - 4.9 [liters/min]** | **ECMO flow > 5.0 [liters/min]** |
| --- | --- | --- | --- | --- |
|  | n = 76 | n = 60 | n =28 | n = 6 |
|  |  |  |  |  |
| **ECMO blood flow [l/min]** | 2.3 ± 0.3 **^a,b,c^** | 3.4 ± 0.2 **^d^** | 4.2 ± 0.2 **^f^** | 6.3 ± 0.5 |
| **ECMO gas flow [l/min]** | 1.8 ± 1.4 **^a,b,c^** | 3.3 ± 1.3 **^e^** | 3.3 ± 0.8 **^f^** | 5.0 ± 0.0 |
| **SV TPTD [ml]** | 78.1 ± 25.1 | 78.1 ± 25.6 | 77.8 ± 22.2 | 95.8 ± 10.3 |
| **SV Echo [ml]** | 76.6 ± 25.0 **^c^** | 76.6 ± 20.5 **^e^** | 81.0 ± 18.5 **^f^** | 117.5 ± 11.4 |
| **Pplat [cm H_2_O]** | 23.0 ± 5.3 | 24.2 ± 6.2 | 23.9 ± 6.4 | 25.5 ± 3.7 |
| **PEEP [cm H_2_O]** | 14.1 ± 2.5 | 13.6 ± 3.1 | 13.0 ± 3.4 | 14.5 ± 1.7 |
| **respirator rate [1/min]** | 12.0 ± 0.0 | 12.0 ± 0.0 | 12.0 ± 0.0 | 12.0 ± 0.0 |
| **tidal volume [ml]** | 141.2 ± 15.4 | 144.3 ± 16.8 | 145.6 ± 19.1 | 127.0 ± 14.0 |
| **heart rate [1/min]** | 90.6 ± 16.3 | 90.6 ± 16.4 | 85.0 ± 14.5 | 95.3 ± 18.4 |
| **systolic RR [mmHg]** | 126.1 ± 23.9 | 126.1 ± 22.3 | 125.3 ± 22.4 | 109.2 ± 9.0 |
| **diastolic RR [mmHg]** | 64.3 ± 11.5 | 64.3 ± 10.6 | 59.8 ± 13.9 | 63.7 ± 6.0 |
| **MAP [mmHg]** | 80.2 ± 16.0 | 80.2 ± 13.1 | 80.3 ± 18.7 | 76.5 ± 4.6 |
| **central venous pressure [mmHg]** | 14.4 ± 5.1 | 14.4 ± 4.3 | 13.4 ± 4.5 | 15.0 ± 1.4 |
| **intraabdominal pressure [mmHg]** | 9.7 ± 3.2 | 9.7 ± 3.0 | 9.4 ± 3.5 | 11.0 ± 2.9 |
| **norepinephrine (µg/kg/min)** | 0.2 ± 0.3 **^c^** | 0.2 ± 0.3 **^e^** | 0.2 ± 0.1 **^f^** | 0.0 ± 0.0 |
| **pH** | 7.4 ± 0.1 | 7.4 ± 0.1 | 7.4 ± 0.1 | 7.4 ± 0.0 |
| **paO2 [mmHg]** | 101.7 ± 27.5 | 101.7 ± 24.3 | 101.2 ± 30.5 | 85.0 ± 19.3 |
| **SaO2 [%]** | 96.7 ± 2.3 | 96.7 ± 4.0 | 97.0 ± 2.5 | 94.8 ± 3.5 |
| **paCO2 [mmHg]** | 52.9 ± 8.0 | 52.9 ± 8.2 | 57.8 ± 15.5 | 57.3 ± 3.5 |
| **lactate (mmol/l)** | 1.3 ± 0.6 | 1.3 ± 1.4 | 1.3 ± 0.6 | 1.1 ± 0.1 |
| **fluid balance** | 4797.7 ± 9187.5 **^c^** | 3147.4 ± 7342.9 | 4373.4 ± 8871.8 f | -2662.3 ± 898.9 |
| **GEDVI (ml/m²)** | 632 ± 137 | 634 ±175 | 645 ±193 | 612 ± 132 |
| **EVLWI (ml/m²)** | 20.6 ± 10.0 | 23.8 ± 10.2 | 24.3 ± 8.8 | 26.4 ± 3.8 |

**Table 2:** physiological data

ECMO, *extracorporeal membrane oxygenation*; SV, *stroke volume*; TPTD, *transpulmonary thermodilution*; Echo, *echography*; Pplat, *plateau pressure*; PEEP, *positive end-expiratory pressure*; RR, *blood pressure*; MAP, *mean arterial pressure*; pH, *negative logarithm of the molar concentration of dissolved hydronium ions in arterial blood;* PaO_2_, *arterial* *partial pressure of oxygen*; SaO_2_, *arterial oxygen saturation*; PaCO_2_, *arterial* *partial pressure of carbon dioxide*; GEDVI, *global end-diastolic volume index*; EVLWI, *extravascular lung water index*

a: ECMO Flow 2.0-2.9 [liters/min] vs. ECMO Flow 3.0-3.9 [liters/min]

b: ECMO Flow 2.0-2.9 [liters/min] vs. ECMO Flow 4.0-4.9 [liters/min]

c: ECMO Flow 2.0-2.9 [liters/min] vs. ECMO Flow > 5.0 [liters/min]

d: ECMO Flow 3.0-3.9 [liters/min] vs. ECMO Flow 4.0-4.9 [liters/min]

e: ECMO Flow 3.0-3.9 [liters/min] vs. ECMO Flow > 5.0 [liters/min]

f: ECMO Flow 4.0-4.9 [liters/min] vs. ECMO Flow > 5.0 [liters/min]
